# Small Heat Shock Proteins: Protein Aggregation Amelioration and Neuro- and Age-Protective Roles

**DOI:** 10.3390/ijms26041525

**Published:** 2025-02-11

**Authors:** Tahani H. Albinhassan, Bothina Mohammed Alharbi, Entissar S. AlSuhaibani, Sameer Mohammad, Shuja Shafi Malik

**Affiliations:** 1 Experimental Medicine Department, King Abdullah International Medical Research Center, King Saud bin Abdulaziz University for Health Sciences, Ministry of National Guard Health Affairs, Riyadh 11426, Saudi Arabia; tahani.hassan1413@gmail.com (T.H.A.); mohammadsa1@mngha.med.sa (S.M.); 2 Zoology Department, College of Science, King Saud University, Riyadh 12372, Saudi Arabia

**Keywords:** proteostasis, proteome, protein-folding, protein aggregation, small heat shock proteins, neurodegenerative disorders, aging, amyloid fibrils

## Abstract

Protein misfolding, aggregation, and aberrant aggregate accumulation play a central role in neurodegenerative disease progression. The proteotoxic factors also govern the aging process to a large extent. Molecular chaperones modulate proteostasis and thereby impact aberrant-protein-induced proteotoxicity. These chaperones have a diverse functional spectrum, including nascent protein folding, misfolded protein sequestration, refolding, or degradation. Small heat shock proteins (sHsps) possess an ATP-independent chaperone-like activity that prevents protein aggregation by keeping target proteins in a folding-competent state to be refolded by ATP-dependent chaperones. Due to their near-universal upregulation and presence in sites of proteotoxic stress like diseased brains, sHsps were considered pathological. However, gene knockdown and overexpression studies have established their protective functions. This review provides an updated overview of the sHsp role in protein aggregation amelioration and highlights evidence for sHsp modulation of neurodegenerative disease-related protein aggregation and aging.

## 1. Challenged Protein Quality Control System

### 1.1. Enormity and Vulnerability of Cellular Proteome

The cellular proteome encompasses the total protein repertoire of a cell expressed at a particular time under specified conditions. The cellular proteome is a highly complex system, gauged from a typical cell expressing around 10,000 genes generating a total copy number of approximately 10^9^–10^11^ molecules [1,2,3]. Another aspect of proteome complexity is protein abundance, with copies per cell varying between 50 copies, in the case of some transcription factors, and millions of copies of histones, ribosomal, and cytoskeletal proteins [4,5]. Although staggering numbers, there are additional components of proteome diversity: protein modifications like methylation, phosphorylation, and acetylation, protein isoforms generated through alternative splicing, the spatial and temporal nature of gene expression, and genome variations [6].

**Figure 1:**
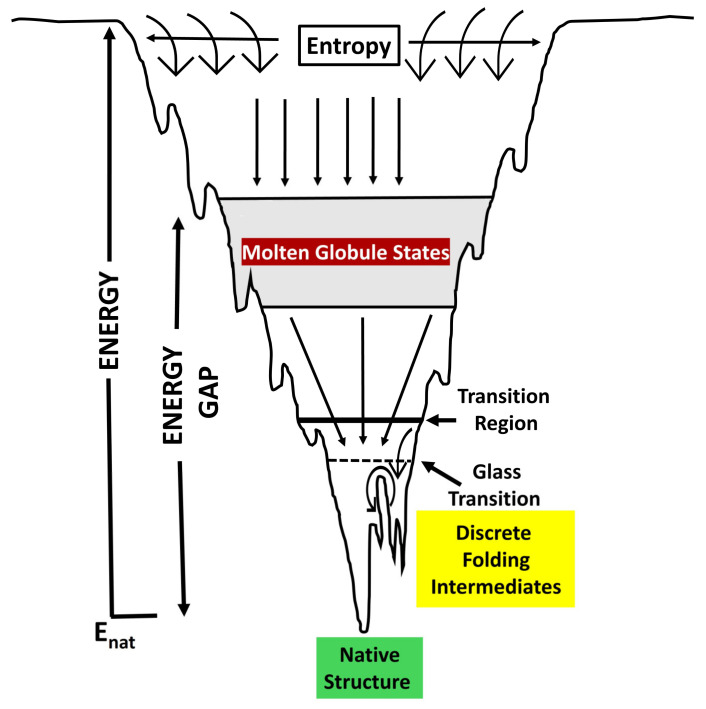
Energy landscape theory of protein folding. The folding process proceeds through the ensemble of conformations starting from the high-entropy upper part of the funnel. The low-energy native structure occupies the base of the funnel.

To perform their biological functions, the newly synthesized proteins must fold into defined three-dimensional structures. Protein folding is an extraordinarily complex self-organizing physiochemical process through which proteins traverse a complex energy landscape. By sampling many conformations in their unfolded state, a thermodynamically favorable unique native fold is attained [7,8,9]. This is defined by the “energy landscape theory” of protein folding (Figure 1) [7,9,10,11]. Protein folding is a function of the inherent amino acid sequence and extraneous contributing factors from the cellular environment [12,13]. The cellular environment is a highly crowded milieu with protein concentrations reaching 50–200 mg/mL [4,5]. This macromolecular crowding restricts entropic freedom for folding polypeptides, thus favoring compact promiscuous interactions and undesired configurations [14]. Consequently, protein synthesis and folding processes are imperfect, and despite quality control mechanisms, around 5–30% of newly synthesized proteins do not fold properly [15,16,17].

Although a folded protein signifies an energetically favored confirmation, most proteins are marginally stable and only correspond to a balance between conformational flexibility required for function and thermodynamic stability [18,19]. This goes well with the notion that proteins essential for critical cellular function are usually structurally dynamic and produced at insufficient soluble levels [20,21]. As a result, proteins are often partially stable in their cellular and physiological environments and therefore prone to folding challenges and consequently needing conformational maintenance [18].

### 1.2. Protein Aggregation

The energy barrier between native and non-native protein conformations is small, consequently restricting the protein energy landscape to a narrow range, meaning proteins are at permanent risk of misfolding. This risk is exacerbated by cellular homeostasis destabilizing conditions like protein native state-disrupting mutations, protein concentration changes, protein structure denaturant presence, or changes in chemical and physical states like pH, ionic strength, and temperature [22,23,24,25,26].

The protein native fold results from the substructural and structural units’ association [27,28,29]. Hydrophobic interactions are at the core of this association and, during the protein folding, have higher exposure propensity. In addition to hydrophobic interactions, protein folding is driven by other interactions like electrostatic, van der Waals, hydrogen bonds, and geometric and steric constraints [7,30]. Protein aggregation is also an outcome of similar interactions, albeit between different molecules, with the critical one being misfolded protein. Protein aggregation and protein folding-degradation pathways are in a kinetic competition tilted towards aggregation by partial folding intermediate presence favoring circumstances [11,15]. Partial unfolding is crucial for misfolded protein formation and globular protein aggregation, exposing the usually shielded hydrophobic regions [31,32]. These partially folded intermediates drive the aggregation process forward by their heavy interactions with and saturation of aggregation-preventing chaperones.

**Figure 2:**
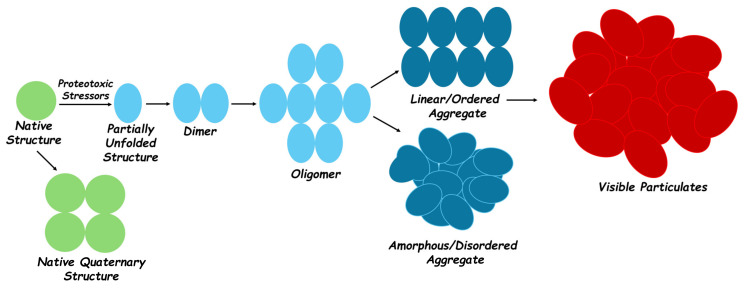
Schematic representation of the protein aggregation process. Proteotoxic stressors—intrinsic, like mutations, and irrelevant, like environmental—generate partially unfolded structures. This initiates the aggregation process, which is an early step in the association of partially misfolded protein molecules. Depending upon the subunit arrangement, aggregates can be linear, like the amyloid type, or more disordered as amorphous aggregates. The larger aggregates have varying degrees of structure and morphology and can be potentially converted into large visible particles.

Protein aggregation initially produces small soluble aggregates through the mutual interactions of misfolded protein molecules or their interactions with other proteins. These small soluble aggregates or, in some cases, amyloid aggregates, also self-assemble from monomeric proteins via their conformationally altered self-complementary surfaces (Figure 2) [33,34]. Covalent bonds, particularly disulfide bonds, further strengthen the oligomeric species and can lead to irreversible aggregates [31,35]. Protein aggregates can be potentially converted into large structures called inclusion bodies [19,36]. Formation of these higher-order compact oligomeric structures is a type of cellular stress response that neutralizes the impact of incorrectly folded toxic proteins by sequestering them in these compact structures, where they await eradication through different mechanisms [37,38]. However, this removes these non-functional proteins from the pool of functionally active proteins. Additionally, these aggregate species might be toxic to cells, which is a common feature in a wide range of neuropathies. Thus, the aggregation process is usually an outcome of extensive cellular damage that leads to cellular coping mechanisms falling short of intracellular refolding and degradative capacity [38,39].

### 1.3. Proteostasis Network

Proteome fidelity, or a state of the balanced proteome, is maintained by proteostasis (*Portmanteau* of protein and homeostasis). Proteostasis ensures coordinated control of concentration, folding, binding interactions, and localization of individual proteins at cellular, tissue, organ, organ system, or organismal levels [18]. At the proteostasis core, the protein must fold into three-dimensional structures for the lifetime maintenance of these properly folded structures [11]. An additional important role of proteostasis is ensuring control over protein abundance, which means the production of correctly folded proteins at the appropriate time, in the correct cellular location, and in quantities that allow proper stoichiometric assembly for oligomeric protein complexes [40]. Proteostasis in human cells is guaranteed through the collective action of approximately 2000 proteins known as the proteostasis network (PN) [18,41]. Achieving its functional optima, proteostasis requires functional coordination between three interlaced arms of the proteostasis network: *protein synthesis and folding*, *conformational maintenance*, and *protein degradation* [19,42]. It guarantees attaining protein quality control goals, ensuring proper protein functioning, preventing damaged protein accumulation, and promoting elimination.

## 2. Protein Aggregation in Disease and Aging

### 2.1. Protein Aggregation and Neurodegenerative Disorders (NDDs)

Due to their post-mitotic nature and cellular structure, neurons are damage-vulnerable to protein aggregation. The post-mitotic characteristic renders neurons devoid of the cell division capacity and hinders their ability to dilute misfolded proteins and associated waste [42,43]. Neurons possess a unique cellular structure: they are highly polarized with long extensions and require unhindered axonal transport between the cell body and the synaptic terminal for adequate functional sustenance [42]. Thus, any physical obstructions to axonal transport, like through protein aggregation, make them highly vulnerable. Therefore, it is not a surprise that the most remarkable common mechanism of neurodegenerative disorders is protein misfolding and aggregate accumulation, making protein aggregation a pathological marker of some neurodegenerative diseases [44,45]. Evidence from genetic, biochemical, cellular, and neuropathological studies in vivo models and post-mortem imaging of brain tissues highlights the significance of protein misfolding and aggregation as triggers for neurodegenerative-disorder-associated pathologies [44,45,46]. In addition to aggregation as the common denominator, NDDs share common features, including spatial neuron destruction, synaptic network connection damage, selective brain mass loss, chronic and progressive disease spread, and enhanced prevalence association with age. Spatial neuron loss defines each NDD and eventually establishes disease-specific clinical manifestations.

**Figure 3:**
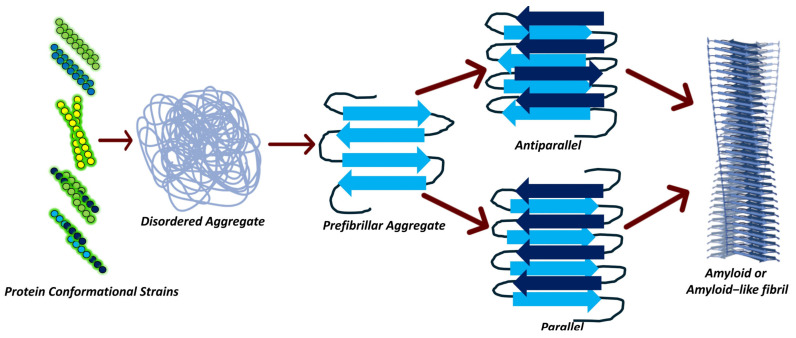
Aggregate formation and cross-β structures. Multiple protein conformations are substrates for aggregate formation. Aggregates are structurally diverse, ranging from unstructured and disordered aggregates to prefibrillar species. Prefibrillar aggregates lead to highly ordered β-sheets containing amyloid fibrils. β-sheets, despite their structural heterogeneity, are stacked in amyloid fibrils.

Notably, the NDD-associated proteins do not possess significant similarities in sequence, size, structure, expression, or function (Table 1). A common theme basic to protein aggregation in neurodegenerative diseases such as Alzheimer’s disease (AD), amyotrophic lateral sclerosis (ALS), familial amyloidotic polyneuropathy (FAP), Huntington’s disease (HD), Parkinson’s disease (PD), and transmissible spongiform encephalopathies (TSEs) is the aggregate-prone mutant proteins. Protein aggregates are a structurally and organizationally diverse entity with cellular toxicity related both to size and level of molecular interactions [47]. The pathological aggregates have three fundamental identities: amorphous aggregates, oligomers, and amyloid fibrils [27]. Based on electron microscopy, protein aggregates are classified into amorphous or amyloid-like fibrils [29]. Protein aggregation variability is best demonstrated by the observed diversity in protein aggregate morphology from the same protein, a consequence of different denaturing conditions [48,49]. Despite these structural and pathophysiological differences among protein aggregate entities, intramolecular β-sheets form a common structural element. In fact, β-sheet formation is the initial step in the NDD protein misfolding. What renders differential traits to these aggregate species is the degree of β-sheet organization, with amyloid fibrils having a higher proportion where the β-sheets run perpendicular to the fibril axis [50,51,52,53,54,55,56]. This β-sheet is also a precursor to amyloid fibril formation (Figure 3). Before the fibril structure formation, β-sheet formation substantially amplifies the protein oligomerization and aggregation. In vitro, amyloid fibrils are formed by β-amyloid (Aβ) and α synuclein proteins, which are associated with AD and PD, respectively [53,54]. The amyloid formation is also amenable to modifications through the chemical milieu of the cellular cytoplasm. For example, the primary extracellular copper form Cu^2+^ inhibits fibril formation [55]. Likewise, calcium modulation of S100A9 self-association and assembly is reported in vitro [56]. It is pertinent to note that not all amyloids are pathological, but physiological ones also exist [57].

Aggregation also has a non-pathological neuroprotective role in which microscopically visible inclusion bodies, an outcome of aggregated protein overproduction, are deposited into a packed structure, an aggresome [58,59]. β-sheets are also critical components of the fibrils that form part of large aggregates and inclusion bodies [60]. Aggresomes are packed inside the microtubule organization center (MTOC), where dynein motors facilitate the cellular aggregate transport along the microtubule network [58]. Inclusion-body-producing neurons displayed improved survival compared to diffuse proteins, with a direct correlation between toxicity and diffuse mutant protein levels [61]. Thus, inclusion bodies are not toxic species but the different oligomeric intermediates that finally lead to forming these inclusion bodies [57].

**Table 1:** The variability in the neurodegenerative disorder-associated proteins.

Disease	Aggregating Protein or Peptide	Structure of Protein or Peptide	Toxicity Mechanism	sHsp Association
Azheimer’s disease (AD)	Amyloid-β peptide	Intrinsically disordered	Gain of toxic function	[62,63,64,65,66,67]
Amyotrophic lateral sclerosis (ALS)	Superoxide dismutase 1 (SOD-1)	β-sheet and IgG-like	Gain of toxic function	[68,69]
Familial amyloidotic polyneuropathy (FAP)	Transthyretin mutants	β-sheet	Gain of toxic function	[70,71]
Huntington’s disease (HD)	Huntingtin with tandem glutamine repeats	Primarily intrinsically disordered	Gain of toxic function	[39,72]
Parkinson’s disease (PD)	α-synuclein	Intrinsically disordered	Gain of toxic function	[73,74,75]
Transmissible spongiform encephalopathies (TSEs)	Prion protein or its fragments	Intrinsically disordered and α-helical	Gain of toxic function	Not reported

### 2.2. Protein Aggregation and Aging

Aging is a time-dependent functional decline driven by the compromised capacity to overcome extrinsic and intrinsic challenges, leading to damage accumulation. One inherent challenge is protein aggregate accumulation, an outcome of the aberrant behavior of the single or multiple factors involved in protein folding and proteostasis maintenance [18,27,76,77].

The nematode *Caenorhabditis elegans* is a model organism for aging research. It has been studied extensively due to its relatively short lifespan and available genetic tools that facilitate aging regulation studies. A few significant and pertinent studies are detailed here. Aging-induced protein insolubilization and enhanced aggregation propensity have been observed in an age-dependent aggregation study [78]. This study identified several hundred aggregation-prone insoluble proteins with a propensity to age-related insolubility increase. A fraction of these insoluble proteins existed at higher levels in older animals, eluding the age-associated tendency of proteins to aggregate. Not all proteins followed similar spatio-temporal insolubility and aggregation trajectories but showed a tissue-specific aging pattern. In addition to this tissue-driven behavior, the spatial pattern of aggregation extends to the subcellular level because of multiple aggregation centers not limited to the aggresome. Furthermore, these aggregation-prone proteins possess distinct structural characteristics like enrichment in aliphatic amino acids and increased β-sheet propensity. These authors further observed the reduction in age-dependent protein insolubility via suppression of insulin/IGF-1 signaling. Another study profiling over 5000 *C. elegans* proteins detected age-associated proteome imbalance through change in abundance in around one-third of the detected proteins [79]. A further important observation is the less pronounced increase in protein levels in the long-lived daf-2 mutant compared to the changes observed in wild-type aging animals. On the contrary, these changes are enhanced in the short-lived daf-16 mutant. In the daf-2 mutant, proteasome abundance is one of the outcomes of the increase in protein levels, which is related to the organismal clearance capacity of surplus proteins. These proteome imbalances are due to age-associated protein translation and turnover changes, accumulating harmful aggregation-prone species. However, protein sequestration in insoluble aggregates is proposed as a protective strategy for proteome integrity maintenance. A study analyzing protein turnover in *C. elegans* models of longevity and aging identified proteome-wide changes over the organism’s life [80]. On day 5 of adult worms at the start of post-reproductive aging, the fastest protein turnover was seen in the longest lifespan strains, compared to the slow turnover observed in the age-related disease model. The fast turnover rate in long-lifespan strains results from protein synthesis and degradation pathways working at high speed, consequently increasing the vulnerability of the protein homeostasis network. Consequently, the authors could not establish a link between protein turnover rates and final adult lifespan.

A multi-modular study analyzed protein misfolding, aggregation, and accumulation in *C. elegans*, senescent cells, and mouse tissues collected at different time intervals from youth to old age [81]. They found a significant accumulation of protein aggregates in aged samples: 1.3–2.5-fold higher insoluble protein than in young samples. These insoluble proteins displayed disease-associated aggregate-like characteristics, including detergent insolubility, protease resistance, and amyloid-binding dye staining, thus establishing a role for protein misfolding and aggregation in aging. Detergent insoluble protein aggregates are also observed to increase markedly and significantly in mouse cardiac tissue with aging and after sustained angiotensin II-induced hypertension [82]. A total of 30% of the aggregated components changed concordantly in both conditions, establishing a relation between protein aggregate accumulation, chronic hypertension, and in vitro myofibroblast senescence. A study comparing skeletal protein aggregation in young and old adults identified a higher abundance of and greater diversity in detergent-insoluble aggregates in aged adults [83]. Ortholog knockdown of some of these abundant proteins in *C. elegans* led to decreased protein aggregation and increased muscle mass, highlighting the commonality in aging-associated genes in nematodes and humans.

## 3. Small Heat Shock Proteins (sHsps)

Molecular chaperones are an essential component of the protein quality control machinery. They form diverse multidomain protein families that facilitate the folding of nascent and newly synthesized proteins, afford protection during proteotoxic stresses, prevent protein aggregation, or assist in the refolding of misfolded proteins [19,84,85]. A molecular chaperone is a protein that interacts with and aids in the folding of other proteins without forming part of their final folded structure [86]. A total of 10% of the cellular proteome is molecular chaperones, and they function during both housekeeping and stress responses [43,87]. A significant proportion of these molecular chaperones are called heat shock proteins (Hsps) because of their stress inducibility during insults like heat shock, oxidative stress, heavy metal, toxic chemical exposure, and inflammation [22,43,88]. Hsps are divided into subgroups based on their size/molecular weight: Hsp40s, Hsp60s, Hsp70s, Hsp90s, Hsp100s, and the small Hsps.

**Figure 4:**
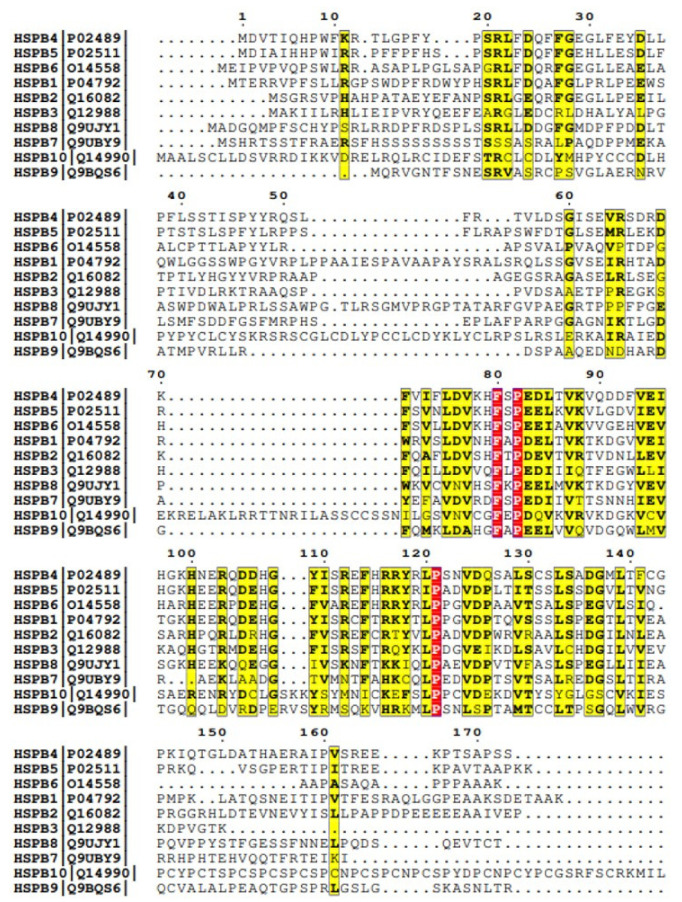
Protein sequence alignment of the ten human sHsps. Alignment was carried out using CLUSTALW [104] and ENDscript [105]. Overall, minimal similarity was observed except in the region harboring ACD. The sequence alignment highlights residue conservation among different sHsps, with color coding indicating the level of conservation.

Small Hsps (sHsps) are a ubiquitous and diverse family of ATP-independent molecular chaperones conserved across species with member expression in all kingdoms of life [22,89]. Their diversity is also in the member numbers: one or two cytosolic members in prokaryotes and unicellular eucaryotes, with an increasing number of members in multicellular eukaryotes [90,91]. The human small HSP family contains ten members (HspB1-B10) that are primarily very divergent at the protein sequence level (Figure 4) and in their functional and associated traits (Table 2) [92,93].

**Table 2:** Human small heat shock proteins.

Protein	M.W (kDa)	Protein Alternative Name	Tissue Distribution	Cellular Localization	Chaperone Function	Stress Inducibility	References
HSPB1	22.8	Heat shock 27 kDa protein (HSP 27)	Ubiquitous	Cytosol, nucleus, cytoplasm, cytoskeleton, spindle, extracellular exosomes	Yes	Yes	[67,94,95,96,97]
HSPB2	20.2	DMPK-binding protein (MKBP)	Heart and muscle	Cytoplasm, cytosol, nucleus		Unknown	[67,96,98]
HSPB3	17	Heat shock 17 kDa protein (HSP 17)	Heart and muscle	Cytoplasm, nucleus, nuclear speck		Unknown	[39,96]
HSPB4	20	Alpha-crystallin A chain (CRYAA)	Eye lens	Cytoplasm, cytosol, nucleoplasm, nucleus	Yes	Yes	[98,99,100,101]
HSPB5	20.2	Alpha-crystallin B chain (CRYAB)	Ubiquitous	Cytoplasm, nucleus, lysosome, cytosol, exosome, cell surface, Golgi apparatus, mitochondria, nucleoplasm	Yes	Yes	[39,67,68,95,96,97,102]
HSPB6	17.1	Heat shock 20 kDa-like protein p20 (HSP20)	Ubiquitous	Cytoplasm, nucleus, nucleolus, cytosol, extracellular region, Golgi apparatus	Yes	Yes	[39,95,96,97,103]
HSPB7	18.6	Cardiovascular heat shock protein (cvHsp)	Heat and muscle	Cytoplasm, nucleus, Cajal body, actin cytoskeleton, nucleoplasm	Yes	Unknown	[95]
HSPB8	21.6	∘ Protein kinase H11 ∘ Small stress protein-like protein HSP22	Ubiquitous	Cytoplasm, cytosol, nucleoplasm, nucleus	Yes	Yes	[39,67,95,96,97]
HSPB9	17.5	Cancer/testis antigen 51 (CT51)	Testis	Cytoplasm, cytosol, nucleoplasm, nucleus		Unknown	
aHSPB10	28.4	Outer dense fiber protein 1	Testis	Nucleus		Unknown	

### 3.1. Primary Structure and Domain Organization

**Figure 5:**
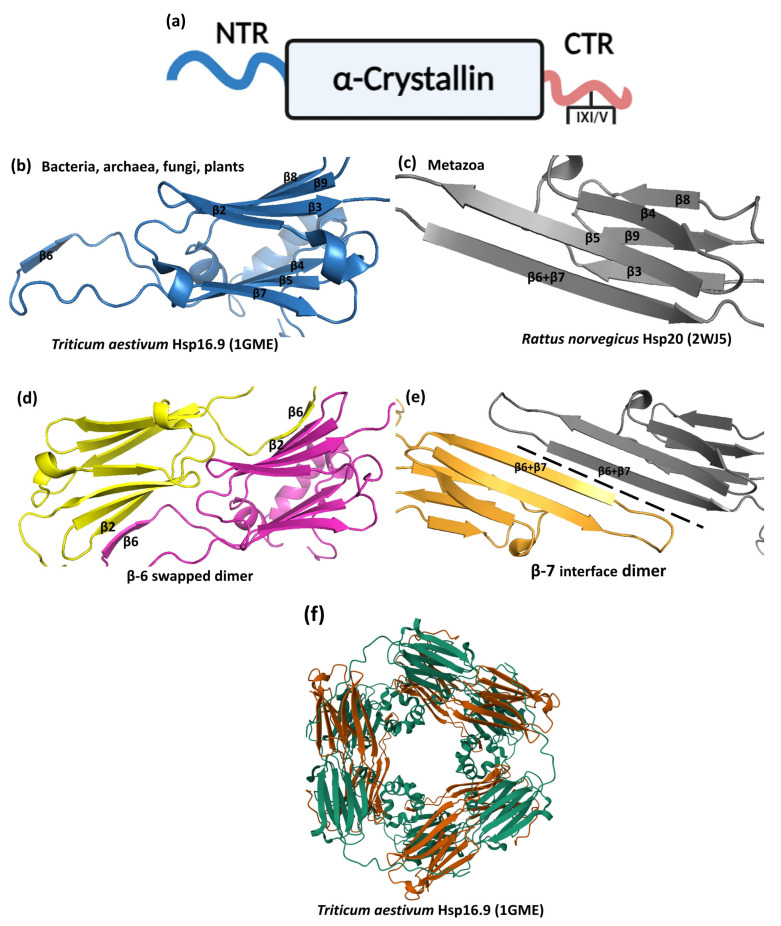
sHsp domain organization, structure, and oligomerization. (**a**) Schematic representation of domain organization. sHsps share the structured α-crystallin domain (ACD), which is flanked by disordered N- and C- terminal regions (NTR and CTR), with the CTR harboring the conserved IX(I/V) motif. (**b**) Bacterial, archaeal, fungal, and plant ACD has a distinct β6 strand in the loop connecting strands 5 and 7. (**c**) Metazoan ACD lacks a distinct β6 strand but contains an extended β7 strand referred to as the β6+β7 strand. (**d**) β6-swapped ACD dimer: in bacteria, archaea, fungi, and plants, a dimer is formed by swapping β6 strands between two ACDs. (**e**) β7 interface ACD dimer: metazoan ACD dimer formation is driven by interactions of the extended β6+β7 strand from the neighboring protomers. A dashed line highlights the interface. (**f**) Higher-order oligomers are formed by ACD dimer cross-linking. This involves ACD-ACD, CTR-CTR, NTR-NTR, and a meshwork of other interactions. [Figure (**a**) was created in BioRender. Malik, S. (2024) BioRender.com/x88u071].

sHsps are defined by low molecular weight (12–43 kDa, average length 160 residues) and tripartite domain architecture. This tripartite domain organization is characterized by a centrally located, conserved, and structured α-crystallin domain (ACD) flanked by variable length, sequence-flexible, and disordered N-terminal region (NTR) and a short C-terminal region (CTR) (Figure 5a) [91,106,107]. The flanking NTR and CTR evolved independently of ACD, setting apart sHsp evolution from other proteins by applying two exon-boundary independent strategies [108].

Structurally, the ACD, the signature motif of sHsps, is a compact 90–100 amino acid residue β-sheet sandwich composed of two layers of three and five antiparallel strands like the immunoglobulin fold (Figure 5b,c) [106,107,109,110]. A short interdomain loop connects the two β-sheet layers.

The NTR, on the other hand, is diverse in sequence and length and consists of 24–247 amino acids (average length 56 amino acids) [91,107]. NTRs are typically rich in hydrophobic residues with an overrepresentation of tryptophans and phenylalnines [111,112]. In the mammalian sHsps, NTR is characterized by phosphorylated serines [113,114]. Unlike ACD, an NTR is a flexible structure concluded from its proteolysis susceptibility [115,116] and high amide proton exchange rates, and amide hydrogen exchange experiments confirm its flexibility [117,118]. NTRs are either partially resolved or completely missing in sHsp crystal structures. However, adapting segments exist within the NTR secondary structure, including in the human αB crystallin [91,107,119].

The CTR, like NTR, is also flexible but shorter in length, <20 amino acids (average length: 10 amino acids) [108]. CTR is polar in character and solvent-exposed, displaying flexibility that increases remarkably towards the C-terminus [120,121]. In most cases (90%), CTR contains the characteristic and conserved three-residue IXI/V motif, which plays a critical role in oligomerization [91,107,122,123]. In a few cases, the IXI/V motif is also present in the N-terminal region of some sHsps, including human HSPB6 [122].

### 3.2. Dimer and Oligomer Assemblies

A remarkable trait of most sHsps, essential for their function, is their oligomerization ability. Most sHsps exist as large, often polydisperse collections, typically composed of 12–32 subunits [124,125]. The sHsp oligomerization proceeds through a hierarchal design. The primary and universal building block of the higher-order sHsp oligomer ensemble is the ACD homodimer [91,107,109,110,118]. ACD dimer formation occurs in either of two ways, based on the β6 strand (β6 loop) involvement of the ACD monomer. In archaea, bacteria, fungi, and plants, the dimer formation proceeds through this β6 strand swapping in which the β6 loop of one ACD interacts with a partnering ACD (Figure 5d) [109,125,126,127]. This dimer structure is commonly referred to as a ‘bacterial’ type dimer with a nomenclature recommendation of ‘β6-swapped dimer’ because of the presence of this conformation in higher eukaryotes (e.g., wheat Hsp16.9; PDB ID 1GME) [120,128]. The other dimer structure, called the ‘metazoan’ type, is a characteristic of metazoan sHsps that do not harbor the β6 loop. These sHsps are characterized by an extended β6-β7 strand formed by the fusion of the β6 and β7 strands into an elongated strand [120,128]. In this ‘metazoan’ type dimer, this β6–β7 strand forms the dimer interface by interacting with the β6–β7 strand of a partnering ACD monomer in a shared antiparallel orientation. Due to the β7 strand involvement, this dimer structure is also called the ‘β7-interface dimer’ (Figure 5e) [125].

The ACD dimer serves as the building block for higher-order oligomerization with both CTR and NTR engagement [124]. More importantly, the conserved IXI motif of the CTR is involved in oligomer formation from the dimers [107,124,125,129,130]. By this IXI motif, the CTR operates as a flexible ‘cross-linker’ between dimers, with the IXI motif of one dimer binding into the hydrophobic groove of the 4 and 8 strands of the neighboring dimer subunit [107]. This bidirectional binding does not essentially proceed through 4 and 8 strands but is also observed for the 8 strand only [109,110,126,131,132,133,134]. This intermolecular IXI-4/8 interaction is considered pivotal to the sHsp oligomer assembly [135]; however, it is also demonstrated that the IX(I/V) disruption does not necessarily disrupt sHsp oligomerization [128,136]. Like CTR, the role of NTR in sHsp oligomer assembly is multi-layered. The NTR of one sHsp dimer is associated with the neighboring dimer through either the ACD or the NTR [91,107]. In *Triticum aestivum* Hsp16.9, the inter-dimer NTR interaction contributes to oligomer formation [109], while as in human HspB2/HspB3, the two sHsp dimers bind to the shared ACD dimer groove of the partnering subunit via an NTR [137]. In addition to these intermolecular contacts, the NTR can also be involved in intramolecular contacts. In the crystal structure of the tapeworm *Taenia saginata Tsp36,* N-terminal region-facilitated dimerization was identified in which the NTR harboring IX(I/V) motif binds into the 4/8 pocket of the partnering subunit [138].

It can be concluded that the ACD dimer is at the core of the sHsp architecture, with multiple labile interfaces, including those from the ACD flanking regions, contributing to oligomerization.

## 4. sHsp Functions

**Figure 6:**
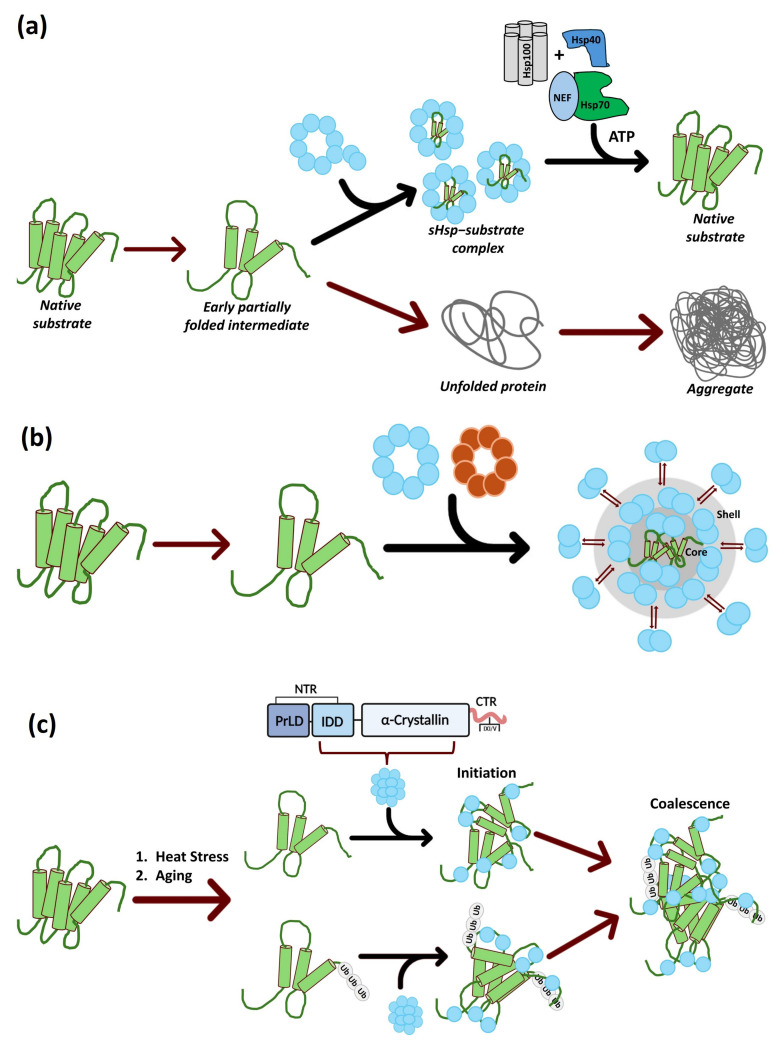
sHsp activity landscape. (**a**) Holdase activity: sHsps bind partially unfolded substrates in an ATP-independent manner to keep them in folding-competent states. The ATP-dependent Hsp70 chaperone system subsequently refolds bound substrates. (**b**) Sequestrase activity: sHsps sequester protein substrates in near-native states in sHsp–substrate complexes in a native-like core structure. sHsp dimers dissociate and reassociate with the sHsp–substrate complex core in a dynamic shell-like ensemble. (**c**) Aggregase activity: pro-aggregation activity of yeast Hsp42 is facilitated by its long NTR composed of a prion-like domain (PrLD) and an intrinsically disordered domain (IDD). Microscopically visible Hsp42–substrate CytoQ/Q-body aggregates coalesce from initially organized aggregates.

sHsps harbor a characteristic chaperone activity distinct from other prominent chaperone families. Mechanistically, sHsps prevent the formation of large, insoluble protein aggregates by binding misfolded or unfolding proteins in an ATP-independent fashion (Figure 6a) [139,140,141]. Usually, sHsps do not possess refolding activities but only stabilize the early unfolding intermediates of aggregation-prone proteins [142], distinguishing these chaperones from ATP-dependent chaperones, which refold unfolded or aggregated protein substrates [85,91]. These sHsp substrates can result from normal physiological processes or proteotoxic stresses like oxidative and heat stress.

sHsp–substrate complexes are a distinct assembly of heterogenous species larger than the substrate-free sHsp oligomers. The substrates in these complexes do not exist as single, soluble protein species. Factors like substrate nature and denaturation conditions determine the architecture and size of these sHsp–substrate complexes [91,107,125,143]. Among these, the sHsp stoichiometry versus substrate protein or the sHsp: substrate ratio is the most critical determinant of sHsp–substrate complex size [102,118,128]. At low sHsp: substrate ratios, sHsps are incorporated in large, dynamic, polydisperse, and insoluble complexes resembling protein aggregates [107,144,145,146]. The difference with aggregates is the sHsp-mediated size reduction and substrate protein conformation alteration [91]. Insoluble sHsp complexes are observed in vivo in many organisms, including in mammalian cells, during heat stress [147,148], and aging in *C. elegans* [24]. As the sHsp: substrate ratio increases, the size of the complex reduces, concomitantly increasing its solubility [91]. These polydisperse and soluble sHsp–substrate complexes have also been observed in vitro [145,149,150,151] and are very heterogeneous; for example, 300 separate complexes can be observed for a 1:1 sHsp: substrate ratio [151]. An important point to note is that in the sHsp–substrate complex, sHsp subunits stay dynamic to fluctuate between bound and dissociated states [146]. This binding and rebinding is not a feature of bound substrates, suggesting the existence of two layers in sHsp–substrate complexes: mobile sHsp constituting the outer shell and sequestered substrate and sHsp forming the immobile inner core [152].

sHsps cannot rescue already unfolded and aggregated substrates; therefore, they must be present during substrate unfolding. For their ATP-independent function, sHsps are termed “holdases” as they capture and hold the unfolded proteins in a protein reservoir competent for post-stress survival disaggregation and refolding [91,107]. This puts sHsps as the first line of defense in the cell that ensures proteome stability under physiological and stress conditions [125]. While sHsps exhibit an aggregation protection role, they are incapable of substrate refolding during stress or resetting physiological conditions. Despite this lack of chaperone activity, substrates are not released spontaneously from the sHsp–substrate complexes, thus placing sHsps in a more sophisticated role as ‘sequestrases’ (Figure 6b) [91,153]. These in vitro sHsp ‘holdase’ and ‘sequestrase’ functions point towards their function as a buffering system in a mechanical manner [91,106,140]; in vivo, functions in a few systems appear to be more pro-aggregation. The yeast sHsp, Hsp42, preferentially associates with peripheral aggregates and is critical to targeting misfolded proteins to these aggregation sites [154]. This aggregate sequestration coupled to site-specific deposition is labeled ‘aggregase’ activity, a cytoprotective protein quality control strategy controlling cytosolic aggregate (CytoQ) formation under stress (Figure 6c) [155]. This cytoprotective activity is specific to Hsp42 because it is not displayed by the other yeast sHsp, Hsp26, which associates both with peripheral and perinuclear aggregates exclusively under heat shock conditions [154]. Hsp42 aggregase activity results from its extended N-terminal domain (NTD), which is critical to targeting misfolded proteins to aggregation sites [154]. One pertinent point is that an Hsp42 homolog is nonexistent in bacteria, plants, or metazoans. Despite the absence of the Hsp42 homolog and putative prion-like sHsp in most organisms, the aggregase activity is not ruled out [155]. As this aggregase activity is NTR-mediated, the generally disordered nature of NTRs and their enrichment in aromatic residues, characteristics like prion-like domains, leaves enough room for aggregase activity in other sHsps [108,155]. Instead of a separation between holdase and aggregase activities, the existence of two diverse activities needs to be interpreted as an outcome of the existence of varied-size sHsp–substrate complex and solubility, specific sHsp features, substrate identity, sHsp: substrate ratio, and the stress type [108,155]. Despite the mechanistic differences, the overarching goal of sHsp-mediated substrate sequestering is packing substrates in a ready-to-refold state for ensuing refolding through ATP-dependent chaperones.

### 4.1. Neuroprotective Functions of sHsps

Progressive accumulation of aggregated proteins into well-ordered structures is a pathological hallmark of neurodegenerative diseases. Knowing that small heat shock proteins are aggregate sequestering chaperones, a protective/functional role is expected. Among the mammalian sHsps, seven sHsps are reported to express in the brain both with spatial- and expression-level connotations: HspB1, HspB2, HspB3, HspB5, HspB6, HspB8, and HspB11 [95,96]. The sHsp expression also displays stress-dependent behavior. HspB1, HspB5, and HspB8 experience heat shock-induced upregulation [96,156]. In addition to their upregulation in heat stress, HspB1, HspB5, and HspB8, along with HspB6, are upregulated during oxidative and hyperosmotic stress in rat hippocampal neurons [157]. Thus, it is evident that sHsps have stress response behavior and hence a neuroprotective function that is extendable to pathological conditions [158]. We discuss the neuroprotective properties of HspB1, HspB5, HspB6, and HspB8 because of their explicit stress-response behavior.

#### 4.1.1. Alzheimer’s Disease (AD)

Alzheimer’s disease (AD) is characterized by pathological lesions, such as senile plaques (SPs), extracellular amyloid plaques (amyloid-beta Aβ-plaques), cerebral amyloid angiopathy (CAA), and intracellular neurofibrillary tangles (NFTs). These predominantly harbor misfolded proteins like hyperphosphorylated tau, hyperphosphorylated neurofilaments, and amyloid-β (Aβ).

HspB1 exists in NFT-containing neurons in a bound state to hyperphosphorylated tau [63,99]. HspB1 differential regulation also correlates with Alzheimer’s disease (AD). HspB1 stained in astrocytes and microglia subpopulations in senile plaques (SPs) and cerebral amyloid angiopathy (CAA) in human brains from AD patients [159]. A previous similar immunohistochemical and immunoblotting analysis revealed the highest HspB1 expression in cortical degenerative astrocytes within the senile plaque (SP)-rich areas [63]. AD is associated with astrocyte reactivity, and HspB1 is a marker for reactive astrocytes. In a study investigating the role of HspB1 in astrocyte–neuron interactions in AD, HspB1 expression in astrocytes is detected with accumulation in glial fibrillary acidic protein (GFAP)-positive astrocytes clustering around amyloid plaques [160]. This study further explored the cell-protective role of HspB1 through heterologous expression. They identified autocrine and paracrine neuroprotective functions, highlighting the therapeutic potential of HspB1 in AD and related tau pathologies. Proteomic profiling of the human AD brain identified HspB1 as one of 58 differentially expressed proteins [64]. In fact, the machine learning approach places HspB1 along with amyloid precursor protein (APP) as a biomarker for Alzheimer’s disease [161].

HspB5 is upregulated in the brain and colocalizes with amyloid-beta in senile plaques. Enhanced HspB5 expression has been detected in AD brains through immunoreactivity in astrocytes, microglia, and oligodendrocytes, pointing toward glial involvement in AD pathology stress response [62,162]. This HspB5 upregulation is correlated with increased NF and tau phosphorylation [159]. In AD brains, a close relationship between HspB5 appearance, extracellular NFTs, and neurofibrillary formation in HspB5-positive neurons has been identified, with a significant relation between the density of HspB5-positive neurons and extracellular neurofibrillary tangles (NFTs) [65]. Likewise, HspB5 has been found to colocalize with amyloid-beta Aβ in the senile plaques [66,67]. That HspB5 colocalization with senile plaques is protective was concluded from physical-activity-enhanced HspB5 expression in the improving hippocampus in an AD mouse model [163]. In vitro studies have identified the HspB5-mediated protection through a direct interaction with Aβ that inhibits Aβ oligomerization from its monomers with additional prevention of oligomer growth into fibrils [67,102,164,165].

HspB6 expression has been reported in cerebral amyloid angiopathy (CAA) in the pathological lesions of AD brains [67]. HspB6-mediated cytoprotection has been reported in cerebrovascular cells through its interaction with Aβ, which inhibits the Aβ-mediated toxicity [67]. The underlying mechanism for this HspB6/Aβ interaction mediated cytoprotection was the reduction in, or complete inhibition of, Aβ aggregation with the HspB6 affinity for Aβ correlated to the degree of cytotoxicity inhibition [166]. HspB6 phosphorylation is reported to enhance the HspB6/Aβ interaction with the phosphor-dependency more pronounced in the case of fibrils than the globular Aβ forms [103].

HspB8 expression has been observed in classic SPs in AD brains and CAAs in hereditary cerebral hemorrhage with amyloidosis-Dutch type (HCHWA-D) brains, another β-amyloid disease [167]. In the WGCNA analysis of the AD hippocampal brain, HspB8 is identified as one of the 10 hub genes contributing to the two AD-specific modular gene networks [168]. Direct interaction between HspB8 and Aβ_1–42_, Aβ_1–40_, and Aβ_1–40_ with the Dutch mutation has been demonstrated along with the HspB8-mediated cerebrovascular cytoprotection [167].

#### 4.1.2. Parkinson’s Disease (PD)

The second most common neurodegenerative disease after Alzheimer’s disease is Parkinson’s disease (PD). Neuronal loss in defined areas of substantia nigra and widespread protein aggregation are the PD hallmarks. α-synuclein, a small (14kDa) intrinsically disordered protein, is a principal constituent of these aggregates. Although neither α-synuclein deposition nor substantia nigra neuronal loss is PD-specific; nonetheless, a combination of the two is considered a conclusive diagnosis [169].

HspB1 mRNA and protein upregulation have been reported in DLB brains as a protective strategy against misfolded and aggregated α-synuclein [170]. This study further reported an 80% HspB1-mediated reduction in α-synuclein-induced toxicity in a culture model. Another study reports the HspB1 overexpression in reactive astrocytes in Parkinson’s disease with dementia (PDD) [74]. The increased HspB1 expression in these reactive astrocytes in the hippocampus is associated with neurofibrillary degeneration. HspB1 expression, along with that of Hsp70, has also been reported in Lewy bodies (LBs) in the substantia nigra from four pathological DLB brains highlighting a direction association with α-synuclein [171]. In vitro analysis of HspB1 interaction with α-synuclein identifies an aggregation prevention role through transient interactions and is dependent on aggregation kinetics with a faster rate limiting the HspB1 preventive function [172]. This HspB1 binding to α-synuclein has been further demonstrated to inhibit fibril growth by elongation prevention [173]. HspB1 overexpression is associated with a significant reduction in intracellular α-synuclein aggregation, highlighting the potential for HspB1-based therapeutic interventions [100].

HspB5 has been reported to be markedly upregulated in the substantia nigra of Parkinson’s disease brain as well as in astrocytes and microglia in a neurotoxin-induced mouse model of Parkinson’s disease [75]. A culture model evaluating the αB-crystallin impact on α-synuclein-induced toxicity reported a 20% HspB5-induced reduction [170]. HspB5, along with HspB1, was reported to be overexpressed in reactive astrocytes in Parkinson’s disease with dementia (PDD), with HspB5 expression also limited to microglia cells [74]. In vitro HspB5-αSyn interaction analysis identifies a transient interaction-mediated aggregation prevention role in which a faster aggregation rate (shorter lag phase) impacts the fibril formation inhibition [172]. HspB5 overexpression leads to a significant reduction in intracellular α-synuclein aggregation; thus, HspB5 overexpression might be a potential preventive way of controlling α-synuclein amyloid fibrillar aggregation [100].

There are not many studies regarding the HspB6 role in Parkinson’s disease. A recent study has demonstrated lipid-dependent chaperone activity in HspB6 [174]. They also found that HspB6 completely inhibited α-synuclein lipid-induced aggregation, with phosphorylation further enhancing this aggregation inhibition capacity. Mechanistically, this lipid-mediated chaperone activity of HspB6 involves a transient complex formation with α-Syn and lipid molecules, thereby decreasing the α-Syn binding affinity for lipids, which delays fibril growth. The lipid dependence of chaperone activity fits well with the progression of α-Synuclein aggregation nucleation through liquid–liquid phase separation (LLPS) in which the α-Syn liquid-like droplets undergo a liquid-to-solid transition to form an oligomer and fibrillar species forming a hydrogel [175].

Like HspB6, HspB8’s role in Parkinson’s disease has not been the subject of many studies. A study investigating the α-Syn binding and aggregation inhibition of sHsps found HspB8 to be the most potent in inhibiting mature fibril formation of wild-type and mutant α-Syn [98]. This high aggregation-inhibiting potency was observed despite the high dissociation constant (*K_D_*) and dissociation rate constant (*k_d_*), which led the authors to suggest a transient but efficient HspB8/α-Syn interaction, efficient enough to prevent α-Syn aggregation.

#### 4.1.3. Amyotrophic Lateral Sclerosis (ALS)

Amyotrophic lateral sclerosis (ALS), or Lou Gehrig’s disease, is another neurodegenerative disease that is a fatal motor neuron disease in which protein aggregates accumulate in the cytoplasm of degenerating neurons and glia as ubiquitin-positive inclusions [176]. TAR-DNA-binding protein 43 (TDP-43) is a significant constituent of these inclusions except in patients with mutations in SOD1 and FUS [177]. Superoxide dismutase 1 (SOD1) is linked to both sporadic and familial ALS [178,179,180]. Genetic screening has identified 13 different ALS families associated with 11 different SOD1 missense mutations, a potential toxic gain-of-function cause of the disease [178]. ALS and FUS are related through gene mutations and abnormal FUS aggregation [179,181].

HspB1 upregulation is reported in an animal model of amyotrophic lateral sclerosis (ALS), with higher expressing HspB1 detected in the nucleus of neurons and glial cells of the spinal cord ventral horn in the early stages, which is localized to reactive glial cells in the later stages [182]. In another ALS animal model with Leu126delTT mutation in the SOD1 gene, an increased HspB1 expression was observed in the reactive astrocytes at the ventral horn of the spinal cord [183]. The relation between SOD1 mutations and HspB1 expression is attributed to the anti-apoptotic and reactive oxygen species (ROS) countering the role of HspB1 [184]. The ability of HspB1 to inhibit SOD1 aggregation in vitro has been demonstrated [101].

HspB5 co-fractionation with insoluble SOD1 entities that include full-length SOD1 proteins, peptide fragments, stable oligomers, and ubiquitinated species have been reported in an ALS mouse model [185]. Detergent insoluble accumulation of the SOD1-L126Z mutant in somatodendritic compartments has been reported in a transgenic ALS mouse model [186]. High HspB5 immunoreactivity has been reported in oligodendrocytes and upregulated expression in astrocytes of symptomatic mice. In the same study, in a cell-free assay, HspB5 suppressed the in vitro aggregation of SOD1-L126Z. HspB5 was identified as the second most abundant protein in hSOD1-immunoreactive inclusions in the transgenic ALS mouse models [187]. HspB5 inhibited SOD1 aggregation in vitro as observed through suppression of SOD1-aggregation-associated thioflavin T fluorescence, and this was attributed to inhibition of aggregate growth [102].

HspB6’s role in ALS is not fully established, and few studies are available. HspB6 is reported to be elevated in activated astrocytes in brain regions of ALS patients [68]. But HspB6 overexpression is conspicuously absent in the spinal cord neuronal cells in ALS patients [68].

HspB8 mRNA levels were significantly increased in the spinal cords of a large cohort of ALS patients, with a remarkable increase in the slower-progressing cases, pointing towards a potential protective role [188]. In a study involving ALS in motoneuron cells, the HspB8 introduction abolished mutSOD1 and TDP-43 aggregation by increasing autophagic clearance [189]. This HspB8 function proceeds through the activation of autophagic flux. It involves the recruitment of the co-chaperone Bag3 (Bcl-2-associated athanogene 3), the chaperone Hsc70 (heat shock cognate 70 stress protein), and the ubiquitinating enzyme CHIP (C-terminus of the Hsc70-interacting protein). This study also reports the high HspB8 levels in end-stage-disease surviving motoneurons from the G93A-SOD1 mice, augmenting the evidence for HspB8 protective function [190]. Potentiating HSPB8-BAG3 interaction is suggested as a target for proteostasis maintenance that can delay ALS progression [191]. The HspB8-mediated autophagic clearance of aggregates has also been reported in a study analyzing the clearance of TDP-43 and its variant TDP-25 aggregates from the nucleus and cytoplasm, respectively [192]. The HspB8 prodegradative activity was also found to be abolished by autophagy inhibitor 3-MA. In the same study, colchicine and doxorubicin enhanced HspB8 expression and led to a reduction in GFP-tagged insoluble TDP-43 and TDP-25, with doxorubicin being particularly active on the GFP-TDP-25.

Table 3 summarizes these sHsp neuroprotective functions. We would also like to refer the readers to some comprehensive review articles covering the other small heat shock proteins and various other neurological disorders [180,193,194,195,196].

### 4.2. Age-Protective Functions of sHsps

Like neurological disorders, aging is accompanied by a progressive decline in proteome quality, with age-associated loss of proteostasis compounding the challenge further. Consequently, the aging proteome is characterized by extensive alterations [79]. One component of compromised proteostasis is the reduced affinity of HSF1 for Hsp genes [197]; nonetheless, age-related enhanced expression of Hsps, including sHsps, has been observed.

A study analyzing heat shock factor-1 (HSF-1) and daf-16 roles in *Caenorhabditis elegans* aging identified a direct relation between reduced activity of these transcription factors and accelerated tissue aging [198]. This correlation with aging acceleration is related to the reduced expression of four small heat shock protein (ship) genes: hsp-16.11, hsp-16.49, hsp-12.6, and sip-1 [198]. A proteomics-based study detected age-dependent aggregation in *C. elegans* with a propensity to aggregate in different tissues and cellular compartments [85]. They also report the presence of small heat shock proteins (HSP-16.11, HSP-16.49, and SIP-1) in these aggregate complexes, attributing it to their role in proteostasis maintenance in younger animals and hence the protective function in aggregation-prone proteins [78]. In another study evaluating the age-associated aggregation in aging *C. elegans*, an increased formation of aggregates with small heat shock proteins was observed, with protein sequestering into chaperone-enriched aggregates as a protective strategy to slow proteostasis decline during aging [79].

In *Drosophila melanogaster,* the mitochondrial small heat shock protein Hsp22 has been demonstrated to display an age-protective function. Ubiquitous or targeted expression of Hsp22 within motor neurons increased the life span by more than 30%, with beneficial effects more prominent in the early aging phase [199]. A follow-up study from the same group reported that the Hsp22 non-expressing flies had a 40% decreased lifespan and died faster than their matched control, along with a 30% decrease in locomotor activity [200]. In oenocytes, Hsp22-based reporters are upregulated dramatically during aging in a cell-specific and cell lineage-specific pattern, which is negatively correlated with the metabolic markers; the probable Hsp22 overexpression is related to aging-induced disruption in mitochondrial protein synthesis [201]. A study analyzed the *Drosophila melanogaster* small HSP family member stress-denatured substrate refolding and disease-associated misfolded protein aggregation prevention capacity [202]. They reported that two sHsp members, one related to acute stress (CG14207) and one to chronic stress (HSP67Bc), extended Drosophila lifespan, thereby highlighting the capacity of functionally diverse sHsps to promote longevity in vivo through different protective approaches [202].

Human fibroblasts increase the basal level of HspB1 (Hsp27) along with some Hsp70 family members in late-passage senescence, indicative of an adaptive response to aging-related intracellular stress [194]. The increase in levels of these proteins is higher in the case of repeated mild heat shock (RMHS) treatment than in normal control cells, and these alterations are correlated to improved functional and survival ability of the cells [191]. A study analyzed the aging-associated sHsp distribution changes in muscle tissues in young and old patients who had undergone orthopedic surgery [203]. Higher expression levels of HspB1 (Hsp27) and HspB5 (αB-crystallin) in the insoluble fraction of vastus lateralis muscles in aged patients were observed, which might limit cytoskeletal disruption or aid in the repair of injured structures in aged skeletal muscle. At the same time, the phosphorylation of both HspB1 and HspB5 was elevated in the soluble fraction [203]. A study characterizing the chaperome network during human brain aging detected 32% chaperome repression, corresponding to ATP-dependent chaperones, and 19.5% induction, corresponding to ATP-independent chaperones and co-chaperones. Among the induced chaperome members, sHsp induction consistently ranked as highly correlated with decreasing median age [39].

## 5. Conclusions and Remarks

This paper discusses the principles of protein aggregation and the functional role of small heat shock proteins (sHsps) in aggregation amelioration, with specifics about their neuro- and age-protective roles. The mammalian small heat shock protein (sHsp/HspB) family is a ten-member family (HspB1-HspB10) characterized by different expression profiles and functions. Functional diversity exists even towards protein aggregation resolution, with poor anti-aggregation (e.g., HspB1) to only anti-aggregation function (e.g., HspB8) or specific nuclear chaperone functions (e.g., HspB5). Their expression is correlated with cell survival and protection in neurodegenerative disorders and aging cells, tissues, and organisms. Most studies are from different cellular and animal models, complicating data interpretation. However, more recent studies have undertaken a multi-modular approach and better data-integration approaches that lay a strong foundation for further mechanistic and translational studies. Therapeutic chaperone introduction through mini-chaperones or peptides with chaperone-like activity has demonstrated a potent impact on protein aggregation pathogenies [204,205,206,207]. These mini-chaperones suppress protein aggregation, stabilize mutant proteins, and inhibit apoptosis [208]. Peptide-based therapeutics have been tested in hyperthermia models [209]. Coupled with their specificities is the sHsp propensity to form homo- and hetero-oligomers that generate new substrate binding and recognition interfaces. Targeted in vivo studies are required to decipher the potency of individual sHsps in aggregation reduction and the mechanism of their contribution to phenotypic resolution. These two synergistic aims will lead to clear translational approaches involving the introduction or upregulation of specific sHsps in a tissue-targeted manner.

## Figures and Tables

**Table 3:** Major sHsp functional modulation in prominent neurological disorders.

Neurological Disorder	sHsp Member	Functional Modulation	References
Alzheimer’s disease	HspB1	Cell protection, biomarker	[63,64,99,159,160,161]
	HspB5	Pathological stress response	[65,66,67,74,102,163,164,165]
	HspB6	Cytoprotection	[67,103,166]
	HspB8	Cytoprotection	[167,168]
Parkinson’s disease	HspB1	Cytoprotection	[74,100,170,171,172,173]
	HspB5	Cytoprotection	[74,100,170,171,172,173]
	HspB6	α-synuclein aggregation inhibition	[174]
	HspB8	α-synuclein aggregation inhibition	[98]
Amyotrophic lateral sclerosis (ALS)	HspB1	Anti-apoptotic, anti-oxidative, SOD-1 aggregation inhibition	[101,182,183,184]
	HspB5	Cytoprotection, SOD-1 aggregation inhibition	[101,185,186,187]
	HspB6	Unclear	[67]
	HspB8	Cytoprotection, SOD-1 aggregation inhibition, TDP-43 aggregation inhibition, TDP-43 aggregate clearance	[189,190,192]

## Data Availability

Not applicable.
